# How echinoccocosis affects potential cancer markers in plasma: galectin-3, sN-cadherin and sE-cadherin? a preliminary report

**DOI:** 10.1186/1746-1596-7-17

**Published:** 2012-02-18

**Authors:** Joanna Giebultowicz, Malgorzata Polanska-Plachta, Piotr Wroczynski, Piotr Zaborowski, Jerzy A Polanski

**Affiliations:** 1 Department of Bioanalysis and Drugs Analysis, Faculty of Pharmacy, Medical University of Warsaw, 1 Banacha Street, Warsaw PL-02097, Poland; 2 Second Department of General Vascular Oncology Surgery, Medical University ofWarsaw, 19/28 Stępińska Street, Warsaw PL-00739, Poland; 3 Department of Gastroenterology, Cardiology and Infectious Diseases, Faculty of Basic Medical Sciences, University of Warmia and Mazury, a 30 Warszawska Street, Olsztyn PL-10082, Poland

**Keywords:** Echinococcosis, N-cadherin, E-cadherin, Galectin-3, Liver cancer

## Abstract

**Background:**

An increasing number of publications are suggesting that galectin-3 (Gal-3) and soluble cadherin fragments, such as E-cadherin (sE-CAD) and N-cadherin (sN-CAD), may be considered as cancer markers. Despite the promising results of the studies, there are no data concerning their levels in the plasma of echinococcosis patients. In most cases, echinoccocosis affects the liver, and its symptoms and disease course are very similar to those of liver cancer. The aim of the present study was to observe whether echinococcosis affects the concentration of soluble sN-CAD, sE-CAD fragments and Gal-3 in plasma and to determine which of them could be considered reliable liver cancer markers for further research.

**Methods:**

The concentrations of sN-CAD, sE-CAD and Gal-3 in the EDTA plasma of patients suffering from echinococcosis (*N = 20*), liver cancers (*N = 10*) and healthy subjects (*N = 20*) were measured using the ELISA method.

**Results:**

The plasma concentration of sE-CAD was lower (*p = 0.0381*), and that of Gal-3 higher (*p = 0.0288*), in echinococcosis than in the healthy group. However, only the concentration of sE-CAD differed significantly among the three analysed groups. In echinococcosis there was a correlation between the sE-CAD and CRP levels (*rs = 0.79*; *p = 0.0066*) as well as a correlation between the sE-CAD level and the number of leukocytes (*rs = 0.65*; *p = 0.0210*) in the blood.

**Conclusions:**

Echinococcosis affects the concentration of soluble sE-CAD fragments and Gal-3 in plasma. sE-CAD can be considered as a marker for differentiation between liver cancer and echinoccocossis, a parasitic liver disease similar in symptoms. Further study is required to confirm these preliminary results.

**Virtual Slides:**

The virtual slide(s) for this article can be found here:

http://www.diagnosticpathology.diagnomx.eu/vs/2115657402650448.

## Background

Echinoccocosis is a parasitic disease caused by a cestode belonging to the genus *Echinococcus*. The most important species for medical purposes are *E. granulosus *(cystic echinococcosis) and *E. multilocularis *(alveolar echinococosis). The liver is the most commonly invaded organ (the liver and the lungs represent more than 90% of reported cases). Pathological symptoms such as abdominal pain and/or jaundice are caused by the pressure the enlarged cyst exerts on the liver tissue. There is additionally a palpable mass in the hepatic area [1]. Similar symptoms are registered in patients with liver cancer. Moreover, echinococcosis and liver cancer have a long asymptotic occurrence. Cystic echinococcosis is usually easy to diagnose using an ultrasound image. It is, however, occasionally difficult to distinguish it from other liver tumours. In these cases, other diagnostic tools, such as serological tests, are used. False positive or false negative reactions, however, may still occur [2]. Moreover, echinococcosis is sometimes not considered during medical diagnosis due to the incidence rates of the two diseases. An erroneous diagnosis of a cyst as being a cancer is associated with the risk of its being damaged during surgery. This can result in anaphylactic shock. Proper cancer markers should therefore be able to distinguish between the cancer and the parasitic cyst.

An increasing number of publications are suggesting that galectin-3 (Gal-3) and soluble cadherin fragments, such as E-cadherin (sE-CAD) and N-cadherin (sN-CAD), may be considered as cancer markers. Although the concentration of proper cancer markers should not only distinguish between healthy and cancerous subjects, but also between cancerous subjects and those suffering from other tumour progression diseases such as echinococcosis, there are no data concerning sE-CAD, sN-CAD and Gal-3 levels in the plasma of echinococcosis patients. This disease may sometimes be misdiagnosed as cancer, which can result in patient death.

The aim of the present study was to observe whether echinococcosis affects the concentration of soluble sN-CAD, sE-CAD fragments and Gal-3 in plasma and to determine which of them are suitable for further research as reliable liver cancer markers.

## Methods

### Materials

Mouse anti-N-cadherin antibodies and 3,3',5,5'-Tetramethylbenzidine (TMB) solution for ELISA were purchased from Sigma-Aldrich (Poznan, Poland). Goat anti-mouse antibodies conjugated with horseradish peroxidase were supplied by Jackson ImmunoResearch Laboratories (Biokom, Janki, Poland). Recombinant Human N-Cadherin/Fc Chimera and Quantikine Human sE-Cadherin Immunoassay were obtained from R&D Systems (Biokom, Janki, Poland). The Gal-3 ELISA kit was purchased from BioVendor R&D (Biokom, Janki, Poland) and the hs-CRP kit from Demeditec Diagnostic (Biokom, Janki, Poland).

### Ethics

This study was approved by the Polish Research Ethics Committee (KB 32/2011).

### Apparatus

Photometric measurements were performed using a BioTek Synergy 5 spectrophotometer (Biokom, Janki, Poland).

### Plasma collection

EDTA-plasma samples were obtained from healthy volunteers (*n = 20*) and from subjects suffering from cystic echinococcosis (*n = 20*) as well as primary (hepatocellular carcinoma, cholangiocarcinoma) and secondary (metastatic adenocarcinoma of the colon and metastatic endometrial cancer) liver cancer (*n = 10*), treated at the Warsaw Hospital for Infectious Diseases and at the Czerniakowski Hospital, Medical University of Warsaw. Plasma samples were separated from blood within 1 to 3 h after blood collection and separated by centrifugation at 2500 rpm for 30 min at about 10°C. The separated samples were stored at -70°C until being assayed.

### ELISA methods

The concentration of sE-CAD, Gal-3 and hs-CRP in plasma was determined by sandwich ELISA using commercially available kits. As no commercially available kit to measure plasma sN- CAD concentration was available, measurements were performed using the procedure reported by Derycke for direct ELISA [3]. The ELISA plates were coated with twice-diluted plasma overnight. Mouse monoclonal anti-N-cadherin antibodies (1:200) were the primary antibody and goat anti-mouse antibodies conjugated with horseradish peroxidase (1:5000) were the secondary antibody. TMB was used as a substrate. The absorbance was recorded at 450 nm, with the correction wavelength at 620 nm, after the reaction was quenched with an acidic solution (2 M aq. HCl).

### Statistical analysis

Normal distribution and variance homogeneity of the data (before and after the variables had been transformed into their natural logarithms) were checked using the Shapiro-Wilk test and Leven's test, respectively. When the data were not normally distributed or their variance not homogeneous (*p < 0.05*), the statistical significance evaluation was performed using the Kruscal-Wallis test. Otherwise, the ANOVA test was used. The correlations between the variables were analyzed using Spearman's rank correlation test, and expressed as *rs *(correlation coefficient) and p (*p < 0.05 *were described as significant). Discriminant function analysis was used to determine which variables discriminate between the examined groups. The classification functions were used to predict a categorical dependent variable by one or more variables. Each function gives the formula according to which the classification scores for the analyzed case can be calculated as follows:

Si=ci+wi1×x1+wi2×x2+…+wim×xm

where: *si *-is the score for *i*'th group of 1,2. . .*m *variables; *ci*-constant for the *i*'th group; *wi1*...*wim*-discriminant function coefficients.

Each case is classified into the group with the highest score. Calculations were performed using STATISTICA version 9.1 software.

## Results

### Analyzed group features

The mean CRP level was 5.0 (7.3) mg/dl in the cancerous patients and 4.9 (5.5) mg/dl (*p = 0.59*; NS) in those suffering from echinococcosis. Their leucocytes levels were 10.5 (3.8) and 5.3 (1.6) K/μl (*p = 0.0022*), respectively. The mean age was 64 yrs (range 38-79) in the cancerous group, 48 yrs (range 21-75) in the echinococcosis group, and 41 yrs (range 28-65) in the control group. Occurrence of Echinococcosis was confirmed using ultrasound imaging and serological tests. Cancers were confirmed histopathologically.

### sN-CAD concentration

Average plasma concentration of sN-CAD in the examined groups is summarized in Table 1. Post-hoc testing revealed that the sN-CAD concentration in the plasma of healthy subjects was significantly higher than in the plasma of echinococcosis (*p = 0.0019*) and liver cancer (*p < 0.00001*) patients. The difference between the sN-CAD level in echinococcosis and liver cancer was not significant (*p = 0.26*). There was also no statistically significant correlation between sN-CAD and CRP levels or between the sN-CAD level and the number of leukocytes in the examined groups.

**Table 1:** Average values (mean, median) and dispersion (standard deviation, interquartile range) of soluble N-cadherin, E-cadherin and galectin-3 concentration in the plasma of the examined group

Concentration [ng/ml]	Echinococcosis (n = 20)		Cancer (n = 10)		Healthy (n = 20)	
	
	Mean (SD)	Median (IQR)	Mean (SD)	Median (IQR)	Mean (SD)	Median (IQR)
sN-CAD	94 (40) *^H^	80 (23)	58 (47) *^H^	50 (70)	141 (11) *^EC^	146 (12)

sE-CAD	9 (11)	6.5 (3.2)*^CH^	12.4 (4.6)	11.0 (1.5) *^EH^	8.6 (1.4)	8.4 (2.0) *^EC^

Gal-3	12.5 (6.0)*^CH^	10.4 (8.8)	9.9 (2.7)*^E^	9.1 (4.1)	9.1 (1.5)*^E^	9.8 (2.3)

*- statistically significant differences (*p *< 0.05) between the examined groups (E-echinoccocosis, C-cancer, H-healthy)

### sE-CAD concentration

Average plasma concentration of sE-CAD in the examined groups is summarized in Table 1. Post-hoc testing revealed that the sE-CAD concentration in the plasma of cancerous subjects was significantly higher than in the plasma of echinococcosis (*p = 0.0001*) patients and healthy subjects (*p = 0.0381*). Moreover, in echinococcosis, there was a correlation between the sE-CAD and CRP levels (*rs = 0.79*; *p = 0.0066*) as well as a correlation between the sE-CAD level and the number of leukocytes (*rs = 0.65*; *p = 0.0210*) in the blood.

### Gal-3 concentration

Average plasma concentration of Gal-3 in the examined groups is summarized in Table 1. Post-hoc NIR testing revealed that the Gal-3 concentration in the plasma of echinococcosis patients was higher than that in the plasma of healthy subjects (*p = 0.0211*) and cancerous patients (*p = 0.1151*, NS). There were no significant differences between the concentration of Gal-3 in the plasma of cancerous and healthy subjects (*p = 0.65*, NS). There was also no statistically significant correlation between Gal-3 and CRP levels or between the Gal-3 level and the number of leukocytes in the examined groups.

### Discriminant analysis

Discriminant function analysis showed that by involving data such as the plasma concentration of Gal-3 and sN-CAD, 100% of healthy cases, 71% of echinococcosis cases, and 67% of liver cancer cases could be correctly classified.

The test of dimensionality for discriminant analysis indicated that only the first dimension was statistically significant (*p = 0.00001*). The standardized canonical discriminant function coefficients were -1.06 for sN-CAD and 0.58 for Gal-3 plasma concentration. The mean canonical roots were -1.44 for the healthy subjects, 0.31 for the echinococcosis patients and 1.13 for the cancer patients. The classification function coefficients are summarized in Table 2.

**Table 2:** Classification function coefficients determined using discriminant function analysis

Variable	Echinococcosis	Cancer	Healthy
sN-CAD [ng/ml]	0.045	0.020	0.093

Gal-3 [ng/ml]	0.550	0.488	0.196

Constant	-6.439	-4.292	-8.709

## Discussion

Galectin-3 is reported to be overexpressed in a variety of neoplastic cells and is suggested to be involved in tumour metastasis by, e.g. enhancing adhesion between tumour cells and extracellular matrix, promoting embolization. However, no generalized conclusion on the role of Gal-3 in cancer has yet been established. The lectin is not only produced and secreted into the plasma by tumour tissues, but also by peritumoural inflammatory cells and stromal cells.

Elevated Gal-3 levels in the serum/plasma have been reported in some cancers, e.g., metastatic breast and gastrointestinal cancer. However, decreased levels of this lectin have been observed in metastatic breast, endometrial and ovary carcinomas, while the levels in Non-Hodgkin's lymphoma and melanoma [4] have not been observed to change. Elevated levels of circulating Gal-3 have not only been observed in cancers, but also in chronic inflammations, e.g. inflammatory bowel disease [5], obesity, type 2 diabetes [6], and heart failure [7,8]. The present studies showed that a higher circulating Gal-3 concentration was observed in liver cancer (NS) and echinococcosis patients than in the healthy control group. The results concerning the cancerous patients, even if not significant, seem to be consistent with the results mentioned above [4] as well as with those reported by Matsuda et al. [9], who observed a higher level of the circulating lectin in liver cancer than in the control group and subjects with chronic liver disease. However, the present report would seem to be the first to compare the levels of Gal-3 in the plasma of cancer patients, echinoccosis patients and healthy volunteers. The highest level observed in echinococcosis could be the result of inflammation related to parasite invasion. Nevertheless, liver cancer is also associated with inflammation and exhibited similar CRP levels to, and higher leukocyte levels than, echinococcosis. Factors other than the inflammation that occurs in echinococcosis might therefore influence the Gal-3 level in the plasma.

sE-cadherin is a form, cleaved from E-cadherin by proteases such as MMP-3, MMP-7 [10], plasmin [11], ADAM10 [12], ADAM15 [13], whereas sN-CAD is cleaved by ADAM10 [14], MT1-MMP [15], MT5-MMP [16], MMP-9, MMP-12 [17], and Presenilin 1 [18]. The induction/upregulation of various MMPs (e.g. MMP-2, MMP-3, MMP-7 and/or MMP-9) has been detected in tumourous liver tissues obtained from, e.g. hepatocellular carcinoma (HCC) patients, whereas the expression of plasminogen activators appears to be largely confined to stromal and inflammatory cells. Inflammation also enhances the expression of MMPs, e.g. in endothelial cells, lymphocytes and macrophages. Furthermore, the expression of certain MMPs and plasminogen activators is also increased in liver cells during liver regeneration [19,20].

Elevated sE-Cad was observed in the serum or plasma of patients with gastric carcinoma [21], bladder cancer [22], non-small cell lung cancer [23], melanoma [24], late-stage colorectal carcinoma [5], and hepatocellular carcinoma [25]. However, higher levels of that cadherin have also been found in gastroesophageal reflux disease [26], multiorgan dysfunction [27], endometriosis [28], familial adenomatous polyposis [25], and acute pancreatitis [29]. Higher amounts of the serum sN-CAD have been described in a group of cancerous patients suffering from prostate carcinoma, breast cancer, ovarian cancer, and gastrointestinal carcinoma, and have been mentioned as being present in diabetes and liver cirrhosis (data not shown) as well [3]. The present study exhibited a higher plasma concentration of sE-CAD in the liver cancer group than the healthy group. This is consistent with the results reported by Sayoma et al. [25].

Moreover, the level of the marker was observed to be lower in the echinococcosis group than the healthy control group. This suggests that sE-cadherin would be a valuable marker of HCC not only because of its association with early recurrence and extrahepatic metastasis [25], but also owing to its ability to distinguish between liver cancer and echinococcosis. In the present study, the plasma sN-CAD concentration in liver cancer was also observed to be lower than that in the healthy group. These results are difficult to explain and require further analysis. Moreover, they seem to be inconsistent with the results reported by Derycke et al. [3]. However, there is no information about the presence of liver carcinoma in the cancerous group examined by them.

The lower levels of the plasmas sN-CAD and sE-CAD in echinococossis patients than in healthy subjects could be explained by the ability of *Echinococcus granulosus *to evade certain defence mechanisms by such means as modulating the host's immune system and resisting the host's proteolytic enzymes by dealing efficiently with, e.g. MMP-9 [19,30]. Discriminant analysis revealed that the association of two or more markers significantly increased their efficiency. These results seem to encourage other scientists to combine two or more markers, which are not effective enough when used separately.

## Conclusions

Echinococcosis affects the concentration of soluble sE-CAD fragments and Gal-3 in plasma. sE-CAD can be considered as a marker for differentiating between liver cancer and echinoccocossis, a parasitic liver disease similar in symptoms. Further study is required to confirm these preliminary results.

## Competing interests

The authors declare that they have no competing interests.

## Authors' contributions

All authors participated in the design of the study. MPP applied for and obtained the approval from an ethics committee. MPP and PZ participated in collecting clinical data, performed the clinical diagnosis and samples collecting. JG carried out the ELISA determination, the statistical analysis and drafted the manuscript. MPP, PW, PZ and JP performed reviewing and editing of the manuscript. JP and PW participated in coordination of the experiment. All authors read and approved the final version of manuscript.
